# Changes in Provision of Psychotherapy in the Early Weeks of the COVID-19 Lockdown in Austria

**DOI:** 10.3390/ijerph17113815

**Published:** 2020-05-27

**Authors:** Thomas Probst, Peter Stippl, Christoph Pieh

**Affiliations:** 1 Department for Psychotherapy and Biopsychosocial Health, Danube University Krems, 3500 Krems, Austria; christoph.pieh@donau-uni.ac.at; 2 Austrian Federal Association for Psychotherapy, 1030 Vienna, Austria; peter@stippl.info

**Keywords:** psychotherapy, COVID-19, Public Health

## Abstract

Reducing personal contacts is a central measure against the spreading of the novel coronavirus disease (COVID-19). This troubles mental health, but also mental health care as treatments usually take place in personal contact and switching to remote treatments might be necessary in times of COVID-19. The present study investigated the question how the provision of psychotherapy changed in the early weeks of the COVID-19 lockdown in Austria and whether there were differences between the four therapeutic orientations eligible in Austria (psychodynamic, humanistic, systemic, behavioral). Psychotherapists (*N* = 1547) completed an online survey. They entered their number of patients treated on average per week (in personal contact, via telephone, via Internet) in the early weeks of the COVID-19 lockdown in Austria as well as (retrospectively) in the months before. The number of patients treated on average per week in personal contact decreased (on average 81%; *p* < 0.001), whereas the number of patients treated on average per week via telephone and via Internet increased (on average 979% and 1561%; both *p* < 0.001). Yet, the decrease of psychotherapies through personal contact was not compensated for by increases of remote psychotherapies (*p* < 0.001). No differences between the four therapeutic orientations emerged. Results imply an undersupply of psychotherapy in the COVID-19 lockdown and that further changes are necessary to cover the increased need for timely psychotherapy in times of COVID-19.

## 1. Introduction

To fight the uncontrolled spread of the novel coronavirus disease (COVID-19), measures to reduce personal contacts (e.g., quarantine, isolation, social distancing) are essential and combined measures are more effective [1].

In Austria, measures of the government against COVID-19 became obligatory on 16 March 2020 (COVID-19 lockdown). At the time of the study, there were only five exceptions of the ban to enter public places [2,3,4]. (1) Averting an immediate danger to life, limb, or property. (2) Professional activity (if home-office is not possible). (3) Errands to cover necessary basic needs. (4) Care and assistance for people in need of support. (5) Exercise outdoors (e.g. running, walking) alone and with pets/people living in the same household. A distance of at least 1 meter to other people has to be ensured. Additionally, certain areas in Austria were under quarantine at the time of the study with even more restrictions.

Although essential to prevent the uncontrolled spread of COVID-19, such measures pose a challenge for mental health and mental health care at the same time. Reviews showed that mental health problems increase during quarantine or isolation [5,6]. Correspondingly, recent studies on mental health during COVID-19 reported increased mental health problems [7,8,9,10]. In Austria, mental health has decreased as well; for example, 4% had clinically relevant depression between 2013 and 2015 but around 20% during the COVID-19 lockdown [11,12]. A living systematic review on mental health during COVID-19 was initiated recently [13]. As recently stated by the United Nations (UN) and others, this leads to an increased need for timely mental health care and governments are asked to provide free and easy access to it [14,15,16]. Yet, switching to remote treatment formats might be necessary as the usual treatment format, i.e., treatment in personal (face-to-face) contact, needs to be avoided or is only possible with restrictions (such as masks, distance, etc.) in times of COVID-19 [17,18,19].

“Opportunities to monitor psychosocial needs and deliver support during direct patient encounters in clinical practice are greatly curtailed in this crisis by large-scale home confinement. Psychosocial services, which are increasingly delivered in primary care settings, are being offered by means of telemedicine.” [15].

To empirically evaluate changes in mental health care in times of COVID-19, the present study investigated how the provision of psychotherapy (personal contact, telephone, Internet) changed in the first weeks of the COVID-19 lockdown in Austria as compared to the months before. Moreover, we examined the question whether changes in provision of psychotherapy differed between the four therapeutic orientations eligible in Austria (psychodynamic, humanistic, systemic, behavioral). 

## 2. Methods

### 2.1. Description of Study Participants

In total, *N* = 1547 psychotherapists completed the online survey. They were M = 51.67 (standard deviation (SD) = 9.69) years old, 75.7% were female (compared to 74.1% female in the Austrian list of psychotherapists in March 2020). Participants were registered in the Austrian list of psychotherapists since M = 11.19 (SD = 9.20) years. The distribution of their psychotherapeutic orientations in comparison to the distribution of therapeutic orientations in the Austrian list of psychotherapists (March 2020) was as follows: psychodynamic (% survey sample vs. % list) 20.9% vs. 25.9%, humanistic 46.3% vs. 37.8%, systemic 22.0% vs. 24.3%, behavioral 9.8% vs. 12.0% (not specified for 1% of the survey sample). Psychotherapists with humanistic orientation were, therefore, overrepresented in the survey sample.

### 2.2. Working Tool

An online survey (open from 24 March until 1 April 2020) comprising 79 items in total was designed in Research Electronic Data Capture (REDCap) [20,21]. The following items were analyzed in the current study: Psychotherapists in Austria were asked about their number of patients treated on average per week since the COVID-19 lockdown as well as (retrospectively) in the months before the lockdown. Psychotherapists were asked about the number of patients treated on average per week for psychotherapy in personal contact, for psychotherapy via telephone, and for psychotherapy via Internet. For the statistical analyses, these numbers were set to 0 for the participating psychotherapists stating that they did not practice psychotherapy in the months before/in COVID-19 lockdown. Additionally, psychotherapists were asked about the psychotherapy method they practice. In Austria, there are 23 accredited psychotherapy methods, which can be classified into four orientations (psychodynamic, humanistic, systemic, behavioral). [22] Only the four orientations and not the 23 methods were examined here.

### 2.3. Investigation Strategy

The survey link was sent via e-mail by the first author in cooperation with the Austrian Federal Association for Psychotherapy to psychotherapists registered in the list of psychotherapists of the Austrian Federal Ministry of Social Affairs, Health, Care and Consumer Protection (>9000 psychotherapists registered in March 2020, with ≈6000 psychotherapists providing a valid e-mail address). Participation was voluntary, without incentives. Psychotherapists had to agree to the data protection declaration to start the survey (electronic informed consent). The principles outlined in the Declaration of Helsinki were followed and the ethics committee of the Danube University Krems (Austria) approved the study. 

### 2.4. Statistics

Statistical analyses were performed with SPSS25. *T*-tests for dependent samples were performed with the total sample to investigate changes in the number of patients treated on average per week between the months before COVID-19 lockdown and in the early weeks of COVID-19 lockdown. Another *t*-test for dependent samples was conducted with the total sample to explore whether decreases (COVID-19 lockdown vs. months before COVID-19 lockdown) in the number of patients treated on average per week by face-to-face psychotherapy in personal contact differed from increases (COVID-19 lockdown vs. months before COVID-19 lockdown) in the number of patients treated on average per week by remote psychotherapy (via telephone + Internet). Moreover, repeated measures analyses of variance (RM-ANOVAs) were performed to investigate whether changes in the number of patients treated on average per week (COVID-19 lockdown vs. months before) interacted with the three treatment formats (personal contact, telephone, Internet) and the four therapeutic orientations (psychodynamic, humanistic, systemic, behavioral). In this RM-ANOVA, the number of patients treated on average per week was the dependent variable. There were two within-subject factors, the first was “change” (two levels: in COVID-19 lockdown, months before COVID-19 lockdown) and the second was “format” (three levels: personal contact, telephone, internet). There was also one between-subject factor, i.e., “orientation” (four levels: psychodynamic, humanistic, systemic, behavioral). All main effects (ME) and interaction effects (IE) were examined. The Greenhouse-Geisser corrected values are reported. Significant ME and IE were followed-up by Bonferroni-corrected simple effects tests. All tests were two-tailed and the significance value was set to *p* < 0.05.

## 3. Results

### 3.1. Results for the Total Sample

The combined (personal contact + telephone + Internet) number of patients treated on average per week in the early weeks of the COVID-19 lockdown (M = 10.12 (SD = 9.05)) was significantly lower than the combined (personal contact + telephone + Internet) number of patients treated on average per week in the months before the COVID-19 lockdown (M = 14.04 (SD = 11.32): average decrease 28%, t(1546) = 13.96; *p* < *0*.001. 

The number of patients treated on average per week in personal contact decreased from M = 13.45 (SD = 10.57) to M = 2.60 (SD = 4.75) (average decrease 81%, t(1546) = 38.80; *p* < 0.001), the number of patients treated on average per week via telephone increased from M = 0.42 (SD = 3.01) to M = 4.53 (SD = 5.77) (average increase 979%, t(1546) = −30.65; *p* < 0.001), and the number of patients treated on average per week via Internet increased from M = 0.18 (SD = 1.35) to M = 2.99 (SD = 4.44) (average increase 1561%, t(1546) = −26.42; *p* < 0.001). 

Decreases in number of patients treated on average per week by face-to-face psychotherapy in personal contact were stronger than increases in number of patients treated on average per week by remote psychotherapy (telephone + Internet): t(1546) = 13.96; *p* < 0.001.

### 3.2. Results for the Four Therapeutic Orientations

**Figure 1:**
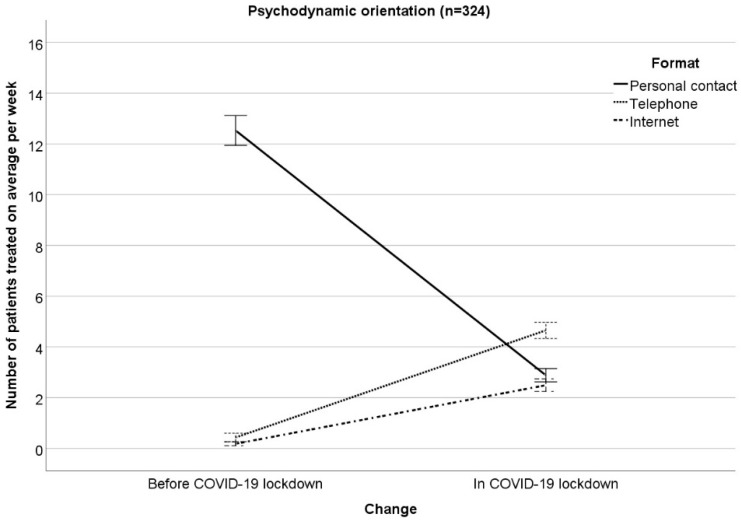
Patients treated on average per week in personal contact, via telephone, via Internet in the months before/in COVID-19 lockdown for psychodynamic orientation. Mean ± 1 standard error.

**Figure 2:**
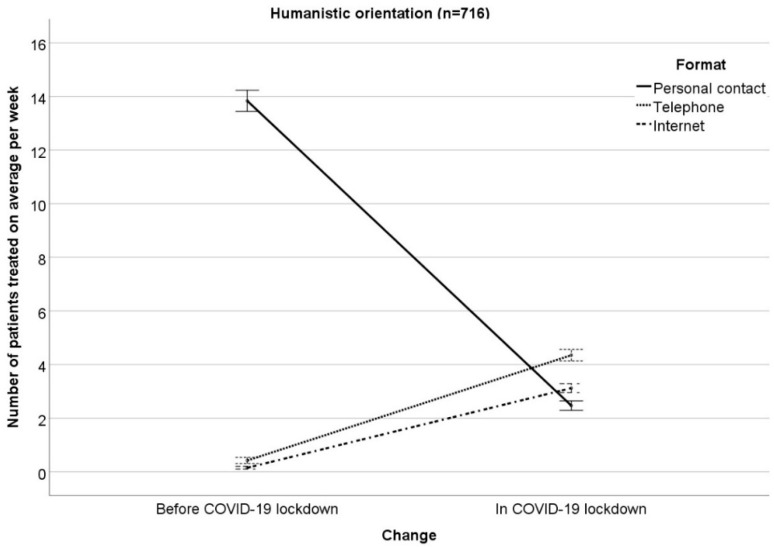
Number of patients treated on average per week in personal contact, via telephone, via Internet in the months before/in COVID-19 lockdown for humanistic orientation. Mean ± 1 standard error.

**Figure 3:**
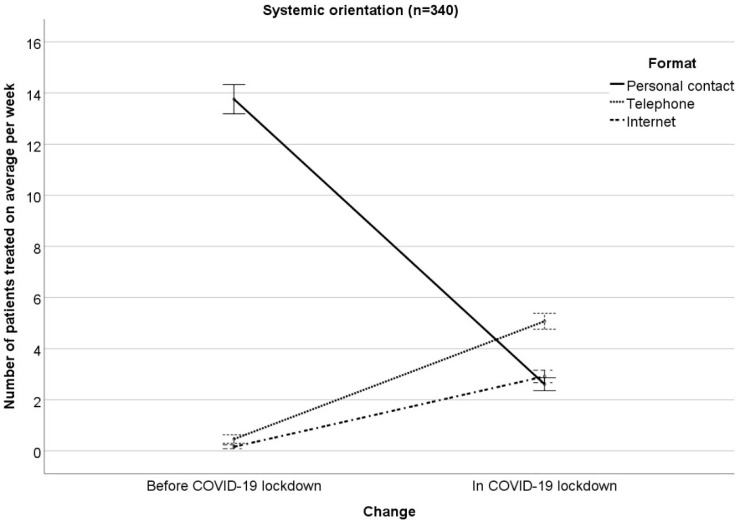
Number of patients treated on average per week in personal contact, via telephone, via Internet in the months before/in COVID-19 lockdown for systemic orientation. Mean ± 1 standard error.

**Figure 4:**
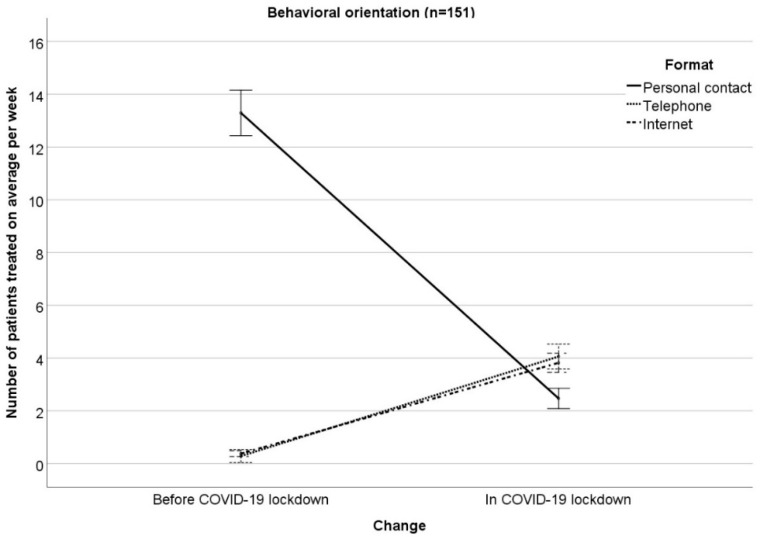
Number of patients treated on average per week in personal contact, via telephone, via Internet in the months before/in COVID-19 outbreak for behavioral orientation. Mean ± 1 standard error.

The results of the RM-ANOVA examining interactions between changes (COVID−19 lockdown vs. months before COVID-19 lockdown) in the number of patients treated on average per week with therapeutic orientation (psychodynamic, humanistic, systemic, behavioral) on the one hand and treatment format (personal contact, telephone, Internet) on the other hand can been seen in Table 1 (Figure 1, Figure 2, Figure 3 and Figure 4 illustrate the results, Figure 1 for psychodynamic therapy, Figure 2 for humanistic psychotherapy, Figure 3 for systemic psychotherapy, Figure 4 for behavioral psychotherapy). Changes in the total number of patients treated on average per week (ME “change”: F(1;1527) = 129.91; *p* < 0.001) were comparable between the therapeutic orientations (IE “change x orientation”: F(3;1527) = 1.21; *p* = 0.304). Changes in the number of patients treated on average per week interacted with treatment format (IE “change x format”: F(1.42; 2166.98) = 1231.43; *p* < 0.001). The ME “format” was significant as well (ME “format” F(1.75; 2665.10) = 829.27; *p* < 0.001). The significant IE “change x format” and the significant ME “format” were followed-up by Bonferroni-corrected simple effects tests.

The Bonferroni corrected simple effects tests for the ME “format” showed that the number of patients treated on average per week was higher in personal contact vs. telephone (*p* < 0.001) and vs. Internet (*p* < 0.001). Moreover, the number of patients treated on average per week was higher for telephone vs. Internet (*p* < 0.001).

For the IE “change x format”, Bonferroni-corrected simple effects tests compared each pair of treatment format at each time point and revealed the following results.

1. Months before COVID-19 lockdown: the number of patients treated on average per week was higher in personal contact vs. telephone (*p* < 0.001) and vs. Internet (*p* < 0.001). Furthermore, the number of patients treated on average per week was comparable for telephone and Internet (*p* = 0.151).
2. In COVID-19 lockdown: the number of patients treated on average per week was lower in personal contact vs. telephone (*p* < 0.001) and vs. Internet (*p* < 0.026). Moreover, the number of patients treated on average per week was higher for telephone vs. Internet (*p* < 0.001).

To summarize, most of the patients were treated in personal contact before the COVID-19 lockdown, whereas most of the patients were treated via telephone in the COVID-19 lockdown.

The reported changes in the number of patients treated on average per week in personal contact, via telephone, or via Internet did not differ between the therapeutic orientations (IE “change × format x orientation”: F(4.26; 2166.98) = 2.19; *p* = 0.063). This means that the therapeutic orientations changed the treatment formats comparably in COVID-19 as compared to the months before. Moreover, the therapeutic orientations were comparable in their number of patients treated on average per week (ME “orientation”: F(3; 1527) = 0.63; *p* = 0.595). In addition, treatment format did not differ between the four therapeutic orientations, i.e., the four orientations treated a comparable number of patients on average per week in personal contact, via telephone, via Internet (IE “format × orientation”: F(5.24; 2665.10) = 1.24; *p* = 0.286).

## 4. Discussion

The COVID-19 lockdown in Austria changed the provision of psychotherapy and this was comparable between the four therapeutic orientations eligible in Austria. Face-to-face psychotherapies in personal contact were reduced and remote psychotherapies (via telephone or via Internet) were increased in the early weeks of the COVID-19 lockdown as compared to the months before. Although average increases in psychotherapies via telephone (979%) or via Internet (1561%) were dramatic, there was an undersupply of psychotherapy in Austria in the early weeks of the COVID-19 lockdown as the total number of patients treated on average per week was lower in COVID-19 lockdown than in the months before. Nevertheless, a positive aspect worth highlighting is the fact that the studied group of psychotherapists was able to maintain a considerable number of patients, those most urgently in need of continuing treatment could be reached by alternatives such as telephone or Internet.

One explanation why increases in psychotherapies via telephone + Internet did not compensate for the decreases in psychotherapies in personal contact might be that the Austrian Internet guideline for psychotherapists valid at the time of the study rejects Internet-based psychotherapy [23]. Psychotherapists might not want to act against this guideline even though health insurances started to cover costs for remote psychotherapy during COVID-19. In this context, it has already been shown that psychotherapists in Austria have neutral to cautious attitudes towards Internet-based psychotherapy [24]. Other reasons might be that psychotherapists perceive too many problems in remote treatments (e.g., technological problems, increased hassle, perceptions of impersonality) [25]. Thus, at least some of them might not be able or might not want to treat remotely. Compared to psychotherapists, individuals with mental health problems have more positive attitudes towards Internet-based psychotherapy [26]. However, at least some patients might prefer to pause sessions until after COVID-19 as the general population does not consider remote psychotherapy as equivalent to psychotherapy in personal contact [27]. Although these findings of previous studies show some reservations against remote psychotherapy, the actual treatment outcome, treatment satisfaction, and the therapeutic alliance can be as good in remote psychotherapies as in psychotherapies in personal contact [28,29,30,31,32]. 

In Austria, health insurance started to financially support psychotherapy via telephone or via Internet during COVID-19 in the same way as they usually support psychotherapy in personal contact. Currently, this is restricted to the COVID-19 situation only but allowing/increasing access to remote psychotherapy in Austria might have several advantages also in the long term when the COVID-19 situation is under control. For example, patients living far away from psychotherapy centers or less mobile patients might benefit. Changes in official guidelines and further adaptations in clinical practice would be necessary to achieve this. In addition, strategies to increase acceptance of remote psychotherapy in the general population might be fruitful [33]. 

The major limitation of the present study is the cross-sectional design. Results refer only to the first weeks of the COVID-19 lockdown in Austria and results might be different some weeks/months later, since initiatives such as crisis helplines and remote psychotherapies for crisis intervention have been started in several regions in Austria. The cross-sectional design also implies that there might be a recall bias regarding the retrospective ratings of the psychotherapists on the number of patients treated on average per week before COVID-19. Multiple measurement points in a longitudinal design would have had more advantages, but the motivation of psychotherapists to take part in such a survey might have been rather low before COVID-19. Moreover, regarding the survey question about the number of patients treated on average per week in the months before COVID-19, no time interval was specified so that different psychotherapists might have given ratings for different time periods. We decided to ask for “months before COVID-19” in general, since psychotherapists can have a stable number of patients treated on average per week over months or even years in Austria. It should also be kept in mind that solely psychotherapists’ self-reports were analyzed and no objective data such as health insurance information. In addition, psychotherapy via Internet was not further specified in the survey and Internet includes several digital media (videoconference, e-mail, chat, …). Furthermore, the results might not be representative for the psychotherapists not participating in the online survey. The participants might, for example, be more used to technology and increases of psychotherapies via telephone or via Internet might be lower in the non-participants. Comparisons with other countries more familiar with e-health in psychotherapy would be interesting, since knowledge and acceptability of digital mental health treatment vary between countries with more or less developed e-health solutions [34].

## 5. Conclusions

To conclude, the COVID-19 lockdown changed the provision of psychotherapy in Austria. While psychotherapy in personal contact was the most often used treatment format before the lockdown, telephone was the most often used format in the lockdown. The practical implications are that further adoptions of mental healthcare are necessary in Austria to cover the need for psychotherapy during COVID-19 and some initiatives have already started. On a scientific level, we could show that the expected decrease in psychotherapies in personal contact were not compensated for by increases in remote psychotherapies in the early weeks of the COVID-19 lockdown in Austria.

## Figures and Tables

**Table 1:** Results of the repeated measures analyses of variance (RM-ANOVAs).

Format	Orientation	Before COVID-19 M (SD)	In COVID-19 M (SD)	Statistics
Personal contact	Psychodynamic *n* = 324	12.53 (9.86)	2.88 (4.86)	ME “change” F(1;1527) = 129.91; *p* < 0.001 IE “change x orientation” F(3;1527) = 1.21; *p* = 0.304 ME “format” F(1.75;2665.10) = 829.27; *p* < 0.001 IE “format x orientation” F(5.24;2665.10) = 1.24; *p* = 0.286 IE “change x format” F(1.42;2166.98) = 1231.43; *p* < 0.001 IE “change x format x orientation” F(4.26; 2166.98) = 2.19; *p* = 0.063 ME “orientation” F(3; 1527) = 0.63; *p* = 0.595
	Humanistic *n* = 716	13.84 (10.94)	2.47 (4.50)	
	Systemic *n* = 340	13.76 (10.75)	2.61 (4.87)	
	Behavioral *n* = 151	13.29 (9.82)	2.47 (5.05)	
Telephone	Psychodynamic *n* = 324	0.43 (2.24)	4.65 (5.06)	
	Humanistic *n* = 716	0.42 (3.85)	4.35 (6.21)	
	Systemic *n* = 340	0.46 (2.22)	5.07 (5.88)	
	Behavioral *n* = 151	0.28 (0.80)	4.06 (4.83)	
Internet	Psychodynamic *n* = 324	0.18 (1.02)	2.49 (3.84)	
	Humanistic *n* = 716	0.15 (0.94)	3.12 (4.57)	
	Systemic *n* = 340	0.16 (0.66)	2.91 (4.19)	
	Behavioral *n* = 151	0.38 (3.35)	3.82 (5.49)	
Total	Psychodynamic *n* = 324	13.14 (10.51)	10.03 (8.44)	
	Humanistic *n* = 716	14.41 (11.87)	9.94 (9.17)	
	Systemic *n* = 340	14.38 (11.36)	10.60 (9.41)	
	Behavioral *n* = 151	13.95 (10.33)	10.35 (8.95)	

Note: SD = Standard deviation; Change = COVID-19 lockdown vs. months before COVID-19 lockdown; ME = Main effect; IE = Interaction effect.
